# Psychosocial areas of worklife and chronic low back pain: a systematic review and meta-analysis

**DOI:** 10.1186/s12891-019-2826-3

**Published:** 2019-10-25

**Authors:** Gabriele Buruck, Anne Tomaschek, Johannes Wendsche, Elke Ochsmann, Denise Dörfel

**Affiliations:** 1 Faculty of Health and Healthcare Sciences, Westsächsische Hochschule Zwickau, University of Applied Sciences, 08012 Zwickau, Germany; 2 Faculty of Psychology, TU Dresden, Dresden, Germany; 3 Division 3 Work and Health, Federal Institute of Occupational Health and Safety, Dresden, Germany; 4 Department of Occupational Medicine, University Medical Center Schleswig-Holstein, Lübeck, Germany

**Keywords:** Areas of work life, Chronic low back pain, Workload, Job control, Social support

## Abstract

**Background:**

The aim of this review was to synthesize the evidence on the potential relationship between psychosocial work factors from the Areas of Worklife (AW) model (workload, job control, social support, reward, fairness, and values) and chronic low back pain (CLBP; unspecific pain in the lumbar region lasting 3 months or longer).

**Methods:**

We conducted a systematic literature search of studies in Medline, PsycINFO, Web of Science, and CINAHL (1987 to 2018). Three authors independently assessed eligibility and quality of studies. In this meta-analysis, we pooled studies’ effect sizes using a random-effects model approach and report sample size weighted mean Odds Ratios (ORs).

**Results:**

Data from 18 studies (*N* = 19,572) was included in the analyses. We found no studies investigating associations between fairness or values and CLBP. CLBP was significantly positively related to workload (OR = 1.32) and significantly negatively related to overall job control (OR = 0.81), decision authority (OR = 0.72), and two measures of social support (ORs = 0.75 to 0.78), even in prospective studies. Skill discretion and reward did not significantly relate to CLBP. Moderation analyses revealed several variables (e.g., exposure time, mean age and sex) affecting these relationships.

**Conclusions:**

Our results support employees’ workload, job control, and social support as predictors of CLBP. In this line, these work factors should be considered when developing programs to prevent chronic low back pain. Future studies should apply measures of CLBP that are more precise, and investigate the full areas of work life (AW) factors in combination.

## Background

An important debate is still ongoing on the relationships between workplace factors and chronic low back pain (CLBP). According to Waddells’ biopsychosocial model of pain [1] chronic pain represents a clinical syndrome that fundamentally differs from acute pain. This distinction applies not only to the duration of the symptoms but also to the presumed causing and maintaining factors of chronic pain, which are supposed to be diverse and include physical, psychological, and social variables. According to this, the model postulates that sensory inputs, cognitive factors, and emotional mechanisms modulate and drive pain development. Empirical findings support the biopsychosocial model: Different social and psychological factors seem to exert considerable influence on the development of chronic back pain [2, 3]. For instance, occupational factors such as employment status, job dissatisfaction, work attitudes, and social support at the workplace have been found to be associated with CLBP [4*, 5–7]. However, information on the consistency of findings and the size of effects is still missing. Data synthesis with systematic reviews or meta-analyses provides the means to shed light on evidence about the antecedents of CLBP.

With a world-wide prevalence of about 23% [8, 9], CLBP is the most prevalent chronic pain condition and severe musculoskeletal disorder. It is associated with high social and economic costs, especially in high-income countries [10]. For instance, CLBP is the leading cause for a premature retirement of employees [11, 12]. Furthermore, CLBP adversely affects the everyday life activities of individuals, their self-perception, and their contact to others [13]. In addition, CLBP is associated with increasing emotional distress and adoption of the sick role [14, 15]. Although there is a great number of studies on the factors driving chronic back pain, a final summary and conclusion of results is difficult as chronic manifestation of pain was not defined consistently throughout these studies [16–18]. Therefore, this work aims to define the outcome more carefully (chronic low back pain) in order to increase comparability between the study results and their validity. We use a specific definition for CLBP that is pain in the lumbar region lasting 3 months or longer. This definition seems to be the most common approach and was used in several studies [19].

In addition to define CLBP precisely, investigating linkages between psychosocial workplace factors and CLBP needs a stronger conceptual and theoretical underpinning in order to increase validity of results. Psychosocial workplace stressors are consistently associated with signs and symptoms of musculoskeletal problems in central body regions and the back [20]. So far, most research on work-related psychosocial risk factors was conducted within the Job-Demands-Control (JDC) framework [21, 22] assuming that high job strain (i.e., jobs characterized by a combination of high job demands and low job control) increases risks for developing low back pain (LBP; e.g. [23, 24]). The review and meta-analysis of Lang, Ochsmann [25] supported this by showing that high job demands (OR = 1.32), low job control (OR = 1.30), high job strain (OR = 1.38), and, in addition, low social support (ORs = 1.19 to 1.42) are associated with increased risks for lower back symptoms. Similarly, Elfering and colleagues [26] found in a longitudinal study that low support from the supervisor increases the risk for LBP. In addition, in the study of Bernal and colleagues [27] effort-reward imbalance was associated with more prevalent musculoskeletal disorders (OR = 6.13) and low social support was related to incidents of back pain (OR = 1.83). In sum, these findings support that LBP in general is related to psychosocial work factors such as high work demands, low job control, low levels of social support and, in addition, low reward. However, whether psychosocial work factors also promote the development of *chronic* pain is still debated [28]. Following this, there is an urgent need to review the literature on CLBP in more detail. Additionally, we consider it necessary to shed light on how the heterogeneous approaches of these studies might impact the findings.

Such a review is also necessary as the working world in western industrialized countries is currently undergoing many changes shaping the workplaces of employees. For instance, digitalization processes might lead to new work tasks and different kinds of work organization [29]. This leads to other work factors related to the health of employees becoming more and more important, for instance, procedural justice and work values [30]. It is therefore the aim of this review to synthesize findings on the associations between these ‘new’ work factors and CLBP, in addition to the “traditional” psychosocial risk factors in occupational health research (demand, control and social support; see [31]). A theoretical approach that integrates such new as well as established psychosocial work factors into a core framework is the Areas of Worklife (AW) model [32]. Based upon an extensive theory and study review, Leiter and Maslach [33] propose that fairness and work values have to be added to workload, job control, social support, and reward [21, 22, 34] when explaining antecedents of job stress, burnout, and work-related strain symptoms more comprehensively [32]. More specifically, fairness refers to how fair and equitable decisions are made within the organization and values concern the fit or conflict between individual and organizational values.

Although the importance of the AW model as six organizational factors was mainly investigated for the development of burnout symptoms [35] there is some initial support for their association with (chronic) low back pain. First, Pohling, Buruck, Jungbauer, and Leiter [36] found that the factors workload, control, reward, and values are related to musculoskeletal complaints. Second, burnout as a unique affective response to chronic exposures of work stress [37] predicts the subsequent development of LBP [38] as well as musculoskeletal pain in several occupational groups [39]. In a large Finnish study [40] burnout was also an important correlate of musculoskeletal disorders among women even after adjusting for other contributing factors.

Therefore, the purpose of our study is to review and quantify the associations between employees’ exposure to the six psychosocial work-related AW factors [32] and CLBP. Our review and meta-analysis adds the following contributions to the literature. In contrast to other reviews [25, 27, 41], we consider the *long-lasting* and *chronic states of lower back pain* as outcome and define CLBP as pain in the lumbar region lasting for 3 months or longer [19]. While the previous reviews investigated associations between LBP and task-related as well as interpersonal work stressors, for instance, job demands, job control, job strain, social support, job security, and monotonous work, we extend this view and add fairness and values as predicting organizational variables.

## Methods

### Criteria for considering studies for the current review

#### Search strategy

The systematic literature search included the following databases: Medline (Pubmed), PsycINFO, Web of Science, and CINAHL. The search strategy was applied to all databases and combined three blocks of keywords: (1) the study population (occupational samples), (2) the outcome (general terms, e.g. musculoskeletal disorders and more sensitive terms, e.g. CLBP), (3) exposure (psychosocial work factors relying on the AW model [32];). Appendix 1 provides the search string. Since the formulation of the new biopsychosocial model of LBP by Waddel [1] launched a new area of publications, the search period started in 1987 and ended in January 2018, Week 3. In addition to the electronic search, reference lists of key review articles were inspected manually.

#### Study selection

**Fig. 1:**
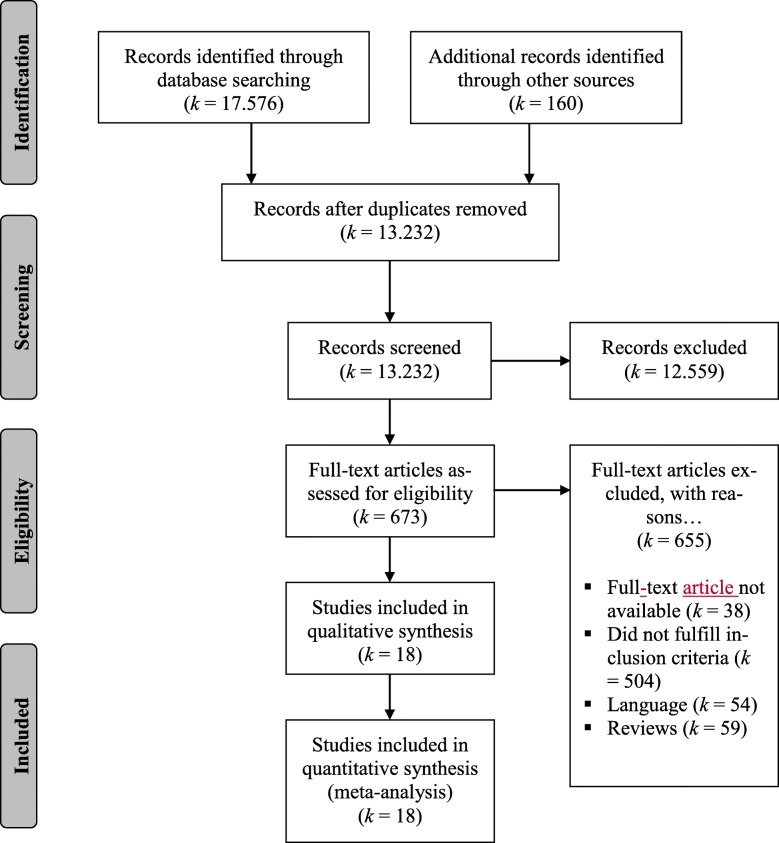
Flow chart of study selection according to PRISMA

Studies were included in the systematic review and the meta-analysis if they were (a) written in English or German and published in a peer-reviewed journal, (b) reported original data on associations between at least one of the psychosocial risk factors included in the AW model (workload, job control, social support, reward, values or fairness) and CLBP (pain in the lumbar region ≥ 3 months; see [19]), and (c) used a sample of working adults (at least 18 years old). After the removal of duplicates, the literature search yielded a total of 13,232 records. All records were reviewed by title and by abstract. Subsequently, three of the authors (GB, DD, AT) conducted a full-text review of 673 records. Finally, 18 studies could be included in the review and meta-analysis. In contrast to the review of Lang et al. [25], which focused on prospective studies only, we also included cross-sectional studies to get further insights on the stability of effect sizes in a further moderator analysis. Figure 1 shows the flow chart of study selection.

#### Quality assessment

Three reviewers independently assessed the methodological quality of the 18 included studies (GB, DD, AT). We used an adapted version of the Scottish Intercollegiate Guidelines Network (SIGN) checklist. To adapt the SIGN checklist, we followed a previous review which analyzed occupational risk factors for musculoskeletal pain or complaints (see [27, 42–44]).

This scale included 8 items grouped into A. study objective/purpose, B. study design/population, C. exposure assessment, D. outcome assessment, and E. analysis and data presentation. See Table 7 in Appendix 2 for all items. Each item was rated as “positive” (when requirement was met), “negative” (when requirement was not met) or “unclear” (unsure if requirement was met). A score was obtained for each study by the sum of all positive responses (1 point each item). Studies were considered as high-quality (++) with 8 positively evaluated items, medium-quality (+) with 6 to 7 positively evaluated items, or low-quality (0) with less than 6 positively evaluated items (adapted from [27]).

#### Data extraction

For each study, we coded the reported effect size and its variance for relationships between psychosocial work factors and CLBP and, in addition, potential moderator variables.

##### Coding of effect sizes

For cross-sectional studies, reported effect size estimates (e.g., correlation *r*, odds ratio OR, risk ratio RR, prevalence ratio PR, or hazard ratio HR) and their variances were extracted. We always used the estimates that were adjusted most comprehensively for confounders. The majority of studies (*k* = 14) reported effect sizes adjusted for age or sex, but only eight studies included both confounders (only age-adjusted: *k* = 5; only sex-adjusted: *k* = 1). For prospective studies, we coded lagged (prospective) effect sizes. If no effect size estimate was reported, we calculated it using other statistical information given in the studies with Comprehensive Meta-Analysis (CMA) software 2.2 (Biostat, Inc., Englewood, NJ). If a relationship was reported as insignificant, but not substantiated by statistical information, the effect size was coded as OR = 1.

##### Coding of moderator variables

The following variables were coded as moderators: year of publication, sample size *N*, occupation (blue, white, pink collar or mixed), country of origin/sampling, study design (cross-sectional or prospective), type of psychosocial work factor, duration of exposure (in months) for prospective studies, samples’ mean age (in years) and sex distribution (percentage of females), and methodological quality of the study (see above).

### Statistical analyses

We used *OR* as effect size measure in this meta-analysis. If studies reported other types of effect size measures they were transformed into *OR*s with CMA software. Reported PRs, RRs, and HRs from prospective studies were considered as equivalent to *OR*s. We are aware that this procedure is warranted only if the incidence of an outcome is low [45]. However, incidences of CLBP in non-exposed groups, which are needed for transformation, were not reported in our sample of studies.

We calculated composite *OR*s whenever multiple associations between constructs of interest were reported. In these cases, we used the mean *OR* and corrected its variance according to the formulas given by Borenstein et al. [46]. In our analyses this occurred for both dimensions of job control (i.e., decision authority and skill discretion) and social support (i.e., from colleagues and from the supervisor). This procedure is necessary because independency of effect sizes is required for pooling [46]. As correlations between dimensions of control and social support were only reported in the study of Eriksen et al. ([47*]; rs = .41) we used them as estimates for correction of variances.

We aggregated effect sizes according to the approach suggested by Hedges and Olkin [48] and calculated sample size weighted mean *OR*s with a random-effects model [46]. We report the number of studies *k*, the cumulative sample size *N*, the sample size weighted mean *OR* and its 95% confidence interval (*CI*). Moreover, we report 95% prediction intervals (PI [46, 49];). In contrast to 95% *CI*s as measure of precision, the 95% *PI* considers the range of effects that may be expected in future studies. Pooled *OR*s with 95% *CI*s excluding zero are significant with *p* < .05.

To estimate heterogeneity in effect sizes, we calculated *Q*- and *I*^2^-statistics [46]. The *I*^2^ indicates the proportion (percentage) of the observed variance in effects across the studies indicating consistency of findings [46]. Values of *I*^2^ ≥ 25% indicate some heterogeneity (25% = low, 50% = moderate, 75% = high), pointing to potential moderator effects. We used subgroup analysis (*Q*-between-statistics) for categorical moderators and mixed effects meta-regression (unrestricted maximum likelihood method) for interval scaled moderators. In line with recommendations of Borenstein et al. [46], we further conducted sensitivity analyses and checked our data for potential publication bias (i.e., inspection of funnel plots, significance of Eggers regression intercept, trim-and-fill-analysis). Moreover, we visualized the study results with forest plots. We conducted all analyses with CMA software 2.2 (Biostat, Inc., Englewood, NJ).

## Results

### Study characteristics

This systematic review aimed at a comprehensive investigation of the relationships between psychosocial work factors, by use of the AW model, and CLBP. However, we only found studies for the following job exposures: workload (*k* = 14), control (*k* = 13), social support (*k* = 12), and only two studies for reward. Therefore, values or fairness as other psychosocial exposures of interest according to the AW model could not be examined. All studies used self-report measures of psychosocial job exposures. Table 1 summarizes the characteristics of the 18 independent studies (*N* = 19,572 employees). Most of them were published between 2000 and 2005 (*k* = 9; 2005–2010: *k* = 6, after 2010: *k* = 4). The median samples size was 634 (*M* = 1087, *Range* = 102 to 7757) with mainly mixed or pink-collar samples (each with *k* = 7; blue collar with *k* = 4). Most studies were of European origin (*k* = 11; Asia: *k* = 4; Australia/New Zealand: *k* = 1; Northern America: *k* = 2). The samples’ mean age was 39 years (range: 32 to 52) with a mean proportion of 52% females. Altogether 10 studies used a prospective design (cross-sectional: *k* = 8). Our quality assessment yielded that most studies had a medium (*k* = 12) or low (*k* = 5) study quality (high: *k* = 1).

**Table 1:** Study characteristics

Reference	*N*	Occupation	Country	Mean age (Years)	Females (%)	Design	Response rate	Exposures	Study quality
Aghilinejad, 2015 [4*]	185	blue collar	Iran	36	0	P	unknown	W + C + S	+
Alexopoulos, 2003 [50*]	351	pink collar	Greece	37	81	CS	90%	W + C (DA) + S (C, SS)	+
Brage, 2007 [51*]	1152	mixed collar	Norway	35	50	P	98% (permission of register follow-up)	C	+
Cameron, 2008 [52*]	303	pink collar	Canada	52	96	CS	61%	C (DA)	–
Elders, 2001 [53*]	288	blue collar	Netherlands	36	0	CS	85% (baseline)	W + C	–
Eriksen, 2004 [54*]	4266	pink collar	Norway	45	96	P	62.3% (baseline) 89.3 (follow-up 1) 85.6 (follow-up 2)	S (S)	+
Eriksen, 2006 [47*]	779	pink collar	Netherlands	40	84	CS	60%	W + C (DA, SD) + S (C, SS)	+
Feng, 2007 [55*]	244	pink collar	China	43	100	CS	91.3%	W + C (DA, SD) + S (C, SS)	+
Hooftman, 2009 [56*]	1259	mixed collar	Netherlands	36	31	P	87% (baseline) 92% (for at least one follow-up)	W + C (SD) + S (C, SS)	+
Hoogendoorn, 2001 [57*]	861	mixed collar	Netherlands	36	30	P	87% (baseline)	W + C (DA, SD) + S (C, SS)	+
Latza, 2002 [58*]	488	blue collar	Germany	32	0	P	85.5% (follow-up) [for baseline not determined]	W + C (DA) + S	+
Matsudaira, 2012 [59*]	836	mixed collar	Japan	44	12	P	86.5% (baseline) 71.6% (follow-up 1) 84.0% (follow-up 2)	W + C (DA, SD) + S (C, SS)	+
Matsudaira, 2015 [60*]	171	blue collar	Japan	42	29	P	86.5% (baseline) 71.8% (follow-up)	R	++
Melloh, 2013 [61*]	169	mixed collar	New Zealand	36	62	P	74% (baseline) 54% (across all follow-ups)	W + S	–
Messing, 2009 [62*]	7757	mixed collar	Canada	36	42	CS	82% / 84% (interviewer-administered / self-administered questionnaire)	W	–
Tsigonia, 2009 [63*]	102	pink collar	Greece	38	93	CS	90%	W + C (SD) + S (C)	+
van den Heuvel, 2004 [64*]	787	mixed collar	Netherlands	36	31	P	87% (baseline)	W + C (DA, SD) + S (C, SS)	+
Violante, 2004 [65*]	858	pink collar	Italy	36	100	CS	95.2%	W + R	–

*N* Sample size, *Age* Mean age in sample, *Females* Percentage of females in sample, *P* Prospective, *CS* Cross-sectional; Exposures assessed: *W* Workload, *C* Control, *DA* Decision authority, *SD* Skill discretion, *S* Social support, *C* Colleague, *SS* Supervisor, *R* Reward, ++ = high, + = medium, – = low

### Meta-analysis

#### AW factors and CLBP

Table 2 shows the pooled *OR*s and heterogeneity statistics. We found that workload significantly and positively related to CLBP with low heterogeneity across the studies. The significant prediction intervals underline this high consistency of effects across studies. Job resources had a protective effect regarding CLBP (*OR*s < 1). However, the effects were only significant for the combined index of job control, for decision authority, and for all social support measures (combined, from colleagues, from supervisor) but not for skill discretion and for reward. Effect sizes for supervisor support showed low heterogeneity (*I*^2^ < .01%), whereas effect sizes for the combined social support measures and for decision authority showed moderate heterogeneity (*I*^2^s: 65 to 73%). Heterogeneity of effect sizes was high (*I*^2^ = 85%) for relationships between reward and CLBP which was also bolstered by insignificant prediction intervals.

**Table 2:** Meta-analytic results for relationships between psychosocial work factors and CLBP

Psychosocial factors	*k*	*N*	OR	95% CI		95% PI		*Q*(*k*-1)	*p*(Q)	*I* ^2^
				*LL*	*UL*	*LL*	*UL*			
Workload	14	14,964	1.32	1.20	1.46	1.20	1.46	13.04	.445	0.3
Job Control										
Combined	13	7635	0.81	0.70	0.94	0.59	1.34	38.56	<.001	68.9
Decision Authority	8	4649	0.72	0.59	0.87	0.39	1.32	25.98	.001	73.1
Skill Discretion	7	4868	0.85	0.70	1.04	0.46	1.56	19.91	.003	69.9
Social Support										
Combined	12	9043	0.77	0.65	0.90	0.46	1.28	32.09	.001	65.7
Colleague	7	4975	0.75	0.61	0.93	0.40	1.40	17.88	.007	66.4
Supervisor	8	8099	0.78	0.70	0.86	0.68	0.89	6.90	.440	<.01
Reward	2	1029	0.67	0.15	3.05	–	–	6.87	.009	85.4

*k* Number of included studies, *N* Cumulated sample size, *OR* Mean sample size-weighted (pooled) odds ratio, *LL* Lower limit, *UL* Upper limit, *CI* Confidence interval, *PI* Prediction interval, *Q* Q-Statistics for heterogeneity and corresponding *p*(Q)-values, *I*^2^ Index of heterogeneity I^2^ (percent)

#### Moderator analyses

**Fig. 2:**
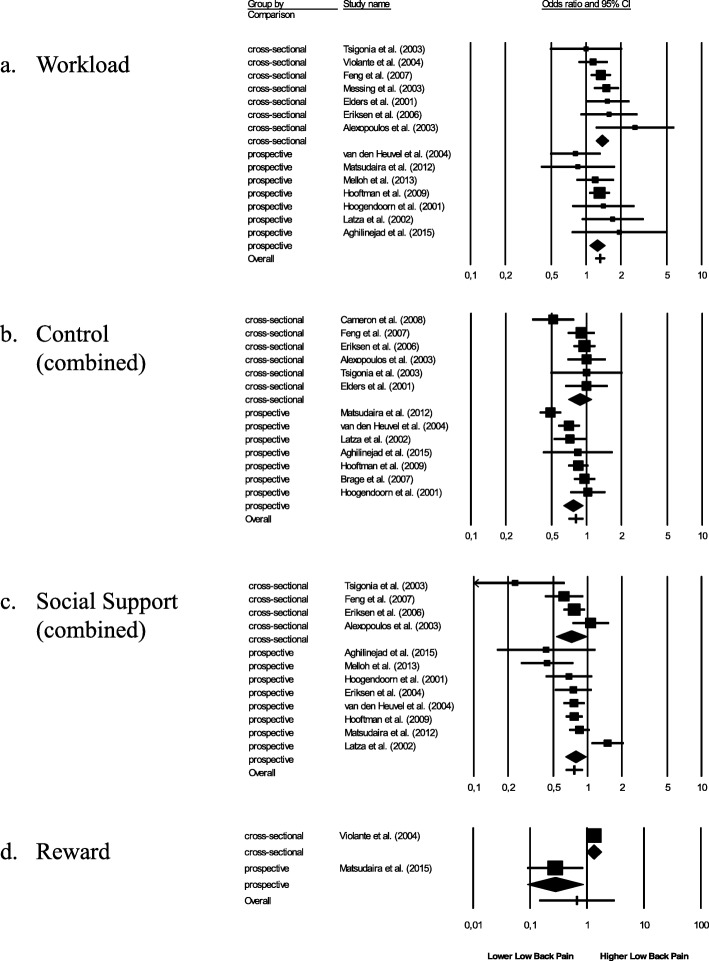
Forest plots for relationships between psychosocial work factors (**a** workload, **b** control, **c** social support, **d** reward) and CLBP for cross-sectional and prospective studies

We conducted a series of moderator analyses to investigate sources of heterogeneity and to check the stability of results further (Tables 3, 4, and 5). Because of low sample size (*k* = 2) moderator analyses for relationships between reward and CLBP were not warranted [46]. First, we found that pooled effect size estimates (*OR*s) were even significant in prospective studies (see Tables 3 and 4 and Fig. 2) with1.25 for workload, 0.77 for job control, 0.63 for decision authority, 0.78 for skill discretion, and 0.78 for social support. However, prospective relationships between colleague support and CLBP were not significant.

**Table 3:** Results of meta-analytic moderator analyses for workload, job control (combined) and job control (decision authority)

	Workload						Job control-combined						Job control-decision authority					
	*k*	OR	*LL*	*UL*	*I* ^2^	z-Test	*k*	OR	*LL*	*UL*	*I* ^2^	z-Test	*k*	OR	*LL*	*UL*	*I* ^2^	z-Test
Study Design																		
Cross-sectional	7	1.38	1.22	1.56	0.0	*p* = .332	6	0.88	0.70	1.10	36.1	*p* = .385	4	0.80	0.63	1.01	52.1	*p* = .164
Prospective	7	1.25	1.07	1.45	2.4		7	0.77	0.63	0.93	77.2		4	0.63	0.49	0.80	68.1	
Occupation																		
Blue	3	1.63	1.16	2.28	0.0	*p* = .441	3	0.84	0.58	1.21	0.0	*p* = .832	1	0.72	0.44	1.19	0.0	*p* = .343
Mixed	6	1.28	1.12	1.47	19.9		5	0.77	0.61	0.97	84.7		3	0.60	0.45	0.81	74.2	
Pink	5	1.32	1.13	1.54	19.0		5	0.85	0.66	1.11	46.9		4	0.80	0.63	1.02	52.1	
Study Quality																		
High	–					*p* = .675	–					*p* = .548	–					*p* = .265
Medium	11	1.30	1.15	1.48	6.3		11	0.82	0.70	0.97	69.9		7	0.74	0.61	0.91	74.1	
Low	3	1.37	1.14	1.64	7.9		2	0.72	0.47	1.10	79.2		1	0.51	0.28	0.95	0.0	
Country																		
Asia	2	1.38	1.04	1.83	0.0	*p* = .946	2	0.89	0.70	1.14	0.0	*p* < .001	1	0.81	0.63	1.04	0.0	*p* = .003
Australia/NZ	1	1.20	0.78	1.84	0.0		–						–					
Europe	9	1.31	1.11	1.54	17.1		9	0.88	0.80	0.96	7.4		5	0.80	0.67	0.95	39.8	
Northern America	2	1.37	1.02	1.86	51.5		2	0.49	0.41	0.60	0.0		2	0.47	0.36	0.62	0.0	
Exposure (months)	7	*PE* = .004, *SE* = .011				*p* = .724	7	*PE* = .001, *SE* = .002				*p* = .346	4	*PE* = −.004, *SE* = .013				*p* = .766
Year of Publication	14	*PE* = −.012, *SE* = .015				*p* = .419	13	*PE* = −.035, *SE* = .017				*p* = .039	8	*PE* = −.056, *SE* = .023				*p* = .014
Sample Size	14	*PE* < .001, *SE* < .001				*p* = .335	13	*PE* < .001, *SE* < .001				*p* = .980	8	*PE* < −.001, *SE* < .001				*p* = .503
Mean Age	14	*PE* = −.004, *SE* = .014				*p* = .798	13	*PE* = −.029, *SE* = .013				*p* = .028	8	*PE* = −.023, *SE* = .016				*p* = .149
Females (%)	14	*PE* = −.001, *SE* = .001				*p* = .645	13	*PE* = .002, *SE* = .002				*p* = .364	8	*PE* = .003, *SE* = .002				*p* = .111

*k* Number of included studies, *OR* Mean sample size-weighted (pooled) odds ratio, *LL* Lower limit of 95% confidence interval (CI), *UL* Upper limit of 95% CI, *I*^2^ Index of heterogeneity *I*^*2*^ (in percent), *PE* Point estimate of predictor from meta-regression (and corresponding standard error SE)

**Table 4:** Results of meta-analytic moderator analyses for job control (skill discretion) and social support (combined and colleagues)

	Job control-skill discretion						Social support-combined						Social support-colleagues					
	*k*	OR	*LL*	*UL*	*I* ^2^	z-Test	*k*	OR	*LL*	*UL*	*I* ^2^	z-Test	*k*	OR	*LL*	*UL*	*I* ^2^	z-Test
Study Design																		
Cross-sectional	3	1.01	0.72	1.43	0.0	*p* = .211	4	0.72	0.53	0.98	69.1	*p* = .651	3	0.68	0.47	1.00	75.9	*p* = .570
Prospective	4	0.78	0.62	0.98	80.7		8	0.78	0.64	0.96	68.3		4	0.78	0.59	1.04	65.2	
Occupation																		
Blue	–					*p =* .211	2	1.21	0.79	1.87	81.6	*p =* .101	–					*p =* .570
Mixed	4	0.78	0.62	0.98	80.7		5	0.73	0.60	0.89	25.0		4	0.78	0.59	1.04	65.2	
Pink	3	1.01	0.72	1.43	0.0		5	0.74	0.58	0.93	58.8		3	0.68	0.47	1.00	75.9	
Study Quality																		
High	–					*p =* 1.00	–					*p =* 1.00	–					*p =* 1.00
Medium	7	0.85	0.69	1.04	69.9		12	0.77	0.65	0.90	65.7		7	0.75	0.61	0.93	66.4	
Low	–						–						–					
Country																		
Asia	1	1.00	0.63	1.58	0.0	*p* < .001	2	0.57	0.34	0.95	0.0	*p =* .212	–					*p =* .057
Australia/NZ	–						1	0.44	0.22	0.87	0.0		–					
Europe	5	0.91	0.81	1.03	0.0		8	0.82	0.68	1.00	70.5		6	0.70	0.58	0.85	47.9	
Northern America	1	0.52	0.41	0.67	0.0		1	0.85	0.53	1.36	0.0		1	1.08	0.73	1.59	0.0	
Exposure (months)	4	*PE =* -.044, *SE* = .012				*p* < .001	8	PE = .022, SE = .006				*p* < .001	4	*PE =* .033, *SE* = .012				*p =* .007
Year of Publication	7	*PE =* −.048, *SE* = .018				*p =* .007	12	*PE =* −.032, *SE* = .020				*p =* .102	7	*PE =* .044, *SE* = .017				*p =* .011
Sample Size	7	PE < −.001, *SE* < .001				*p =* .581	12	PE < .001, *SE* < .001				*p =* .713	7	PE < .001, *SE* < .001				*p =* .620
Mean Age	7	*PE =* −.035, *SE =* .023				*p =* .130	12	*PE =* −.020, *SE =* .019				*p =* .287	7	*PE =* .036, *SE =* .021				*p =* .081
Females (%)	7	*PE =* .005, *SE =* .002				*p =* .033	12	*PE =* −.003, *SE =* .002				*p =* .088	7	*PE =* −.004, *SE =* .003				*p =* .185

*k* Number of included studies, *OR* Mean sample size-weighted (pooled) odds ratio, *LL* Lower limit of 95% confidence (precision) interval (CI), *UL* Upper limit of 95% CI, *I*^2^ Index of heterogeneity I^*2*^ (percent), *PE* Point estimate of predictor from meta-regression (and corresponding standard error SE)

**Table 5:** Results of meta-analytic moderator analyses for social support from supervisor

	*k*	OR	*LL*	*UL*	*I* ^2^	z-Test
Study Design						
Cross-sectional	3	0.84	0.69	1.02	48.9	*p =* .380
Prospective	5	0.76	0.67	0.86	0.0	
Occupation						
Blue	–					*p =* .497
Mixed	4	0.76	0.66	0.87	0.0	
Pink	4	0.82	0.68	0.98	28.4	
Study Quality						
High	–					*p =* 1.00
Medium	8	0.78	0.70	0.86	0.0	
Low	–					
Country						
Asia	1	0.62	0.42	0.91	0.0	*p =* .155
Australia/NZ	–					
Europe	6	0.82	0.73	0.93	0.0	
Northern America	1	0.67	0.52	0.85	0.0	
Exposure (months)	5	*PE =* −.009, *SE =* .010				*p =* .364
Year of Publication	8	*PE =* −.029, *SE =* .017				*p =* .083
Sample Size	8	*PE* < −.001, *SE* < .001				*p =* .751
Mean Age	8	*PE =* −.019, *SE =* .014				*p =* .189
Females (%)	8	*PE =* .001, *SE =* .002				*p =* .494

*k* Number of included studies, *OR* Mean sample size-weighted (pooled) odds ratio, *LL* Lower limit of 95% confidence (precision) interval (CI), *UL* Upper limit of 95% CI, *I*^*2*^ Index of heterogeneity I^*2*^ (percent), *PE* Point estimate of predictor from meta-regression (and corresponding standard error SE)

Occupation, study quality, and sample size were not detected as moderators affecting pooled effect size estimates. For some exposure-CLBP relationships, we found significant differences between countries. However, such effects might be spurious because of the low number of studies in the subcategories. Therefore, an interpretation of effects is not warranted [46].

Regarding the prospective studies, we found that duration of exposure (i.e., time between assessment of work-related factor and CLBP) affected the relationships between CLBP and between skill discretion as well as social support from colleagues (and the combined social support measure). However, the direction of effects was inconsistent. We do not interpret these results as these analyses were based on a low number of studies (4 ≤ *k* ≤ 8 [46];). Year of publication was partially supported as moderator. That means that studies that were more novel reported stronger protective effects for relationships between CLBP and job control as well as colleague support. Moreover, the negative relationship between CLBP and job control was strengthened with an increasing mean age in the samples and the negative relationship between CLBP and skill discretion was strengthened with increasing number of males in the samples.

#### Sensitivity analyses

We also checked for potential outliers across the studies. However, for none of the examined relationships we found evidence for such extreme deviations of single effect sizes (all primary study *OR*s between ±3 *SD* from the mean).

With regard to potential time trends, we conducted a series of cumulative meta-analyses according to Borenstein et al. [46]. For this reason, we sorted and entered the studies effect sizes chronologically for pooling. The forest plots (not shown here) displayed a rather consistent narrowing and stabilization of pooled effect size estimates and their confidence intervals. This means that time of publication per se does not affect the interpretation of our results.

We further examined how significance of the pooled effect size estimates changes if certain studies would be excluded from the analysis (one-study-removed procedure described by [46]). For workload, control, and social support no shift in effect sizes was found. However, with regard to small sample of studies considering reward and CLBP we note that the reported protective effect (*OR* < 1) in the study of Violante et al. [65*] was significant while the effect reported by Matsudaira et al. [60*] was not.

#### Publication bias

A potential publication bias might affect the accuracy of meta-analytic results [46]. We investigated such a systematic neglect of study results as described below. First, funnel plots (can be requested from the authors) did not indicate an asymmetric distribution of effect sizes and standard errors. However, funnel plot analysis is largely based on subjective judgement. Therefore, Table 6 shows some statistical procedures for detecting a publication bias. We conducted a trim-and-fill analysis which simulates pooled effect size estimates under the assumption that (hypothetic) effect sizes are included that bring their total distribution to (nearly) perfect symmetry in the funnel plot. While mean pooled effect size estimates and their significance hardly changed for workload, job control (combined and decision authority), and social support (combined, colleagues, supervisor), the simulation yielded stronger protective effects on CLBP for skill discretion (OR = 0.77 instead of OR = 0.85 from this sample of studies) which was now significant. We further investigated asymmetric effect size distribution with a test of intercepts by Egger. However, intercepts were not significant. Thus, asymmetry was not indicated. In sum, our analyses might indicate a potential (small) publication bias for relationships between skill discretion as dimension of job control and CLBP revealing that our results might underestimate potential true effect size estimates.

**Table 6:** Results of analyses for publication bias

Psychosocial factors	*=k*	Trim & fill (Duval & Tweedie)				Egger test		
		*k*(t)	*OR*(t)	95% CI		*b*	95% CI	
				*LL*	*UL*		*LL*	*UL*
Workload	14	1	1.31	1.16	1.46	0.13	−1.15	1.42
Job Control								
Combined	13	2	0.79	0.68	0.91	0.68	−2.58	3.93
Decision Authority	8	1	0.70	0.58	0.84	−0.52	−5.13	4.08
Skill Discretion	7	2	0.77	0.64	0.93	0.85	−5.25	6.95
Social Support								
Combined	12	4	0.85	0.72	1.01	−1.32	−3.77	1.12
Colleague	7	1	0.79	0.62	0.99	−1.84	−6.13	2.45
Supervisor	8	0	0.78	0.70	0.86	0.52	−2.49	3.54

*k* Number of included studies, *k(t)* Number of trimmed studies, *OR(t)* Estimated OR after including trimmed studies, *CI* Confidence interval, *LL* Lower limit, *UL* Upper limit, *b* Regression intercept

## Discussion

Using data from 18 studies with 19,572 employees in total, this systematic review and meta-analysis examined relationships between psychosocial work factors from the AW model [32] and CLBP lasting 3 months and longer [19]. Our results suggest an overlap between psychosocial workplace factors associated with low back pain in general [25, 27] and those associated with CLBP: Workload, job control, and social support. Therefore, the proposed yellow flags for CLBP should be re-assessed. However, psychosocial factors that Leiter and Maslach [32] suggested as job resources against work stress developing from current changes in the working world, for instance, high fairness and a fit between personal and organizational values, have been widely neglected in relation to CLBP. Future research should strengthen research in these areas to get a more comprehensive and complementary view on how different work-related psychosocial risk factors affect the long-term development of musculoskeletal problems.

### AW factors and CLBP

The results of our review and meta-analysis supported that well-known psychosocial work factors such as workload, job control, and social support significantly relate to CLBP. More specifically, high workload increases the risk whereas high job control reduces the risk for developing CLBP. However, the latter association was supported only for the combined measure of job control and for decision authority alone, but not for skill discretion. High social support from colleagues and supervisors also proved to be a resource that prevents or reduces the risks for CLBP. Our findings contribute to the literature in at least two ways. First, our results extend findings from other meta-analyses in this field of research revealing that high work-related psychosocial risk factors such as high workload, low job control, and low social support not only increase the risk for current musculoskeletal symptoms [25, 27] but also drive the development of CLBP in a long run. Second, our results also bolster theoretical assumptions from the Job-Demands-Control-Support model (JDCS [21];) that these three central work factors not only affect employees’ well-being (see [31] for a review) but also relate to physical strain symptoms. Theoretical models on how psychosocial work stressors affect the development of musculoskeletal strain reactions assume two paths (see [41] for an integration of study results): (a) a physical one via increased load at work and (b) a psychophysiological one via increased muscle tension, prolonged activation of motor units, and changes in blood supply and anabolic activity. Our purpose was not to uncover the exact mechanisms of CLPB. However, this is an important task for future reviews because such information might be helpful in developing preventive measures at the worksite.

The purpose of the AW model [32] was to extend the traditional JDCS model by including new and theoretically-based work factors with a further potential to reduce upcoming strain-reactions from work. One of those, reward, was considered in two studies but the pooled effect size for relationships to CLBP was not significant here. The results of the sensitivity analysis also showed inconsistent associations. We note that such small sized and heterogeneous effects might also be due to ignoring potential moderators such as the level of workload and the individuals’ tendency to work more than expected [34]. These moderators could strengthen the risks of low reward for CLBP. However, such moderating effects were not investigated in our selected sample of studies calling for more research efforts in future. This also concerns the impact of psychosocial risk factor patterns. For instance, Lang et al. [25] and Hauke et al. [41] found some initial support that the risks for back symptoms are significantly increased under high strain jobs, which means a combination of low control and high demands.

We found no studies investigating the relationships between CLBP and fairness and values. Associations between workplace injustice, which means a lack of fairness, and backaches have been reported [66, 67]. However, fairness and values are the motivating connection between the worker and the workplace, which goes beyond the utilitarian exchange of time for money or career. Due to globalization and digitalization those psychosocial work factors become increasingly important [30] and on their relation to physical well-being should be more concentrated in the future.

In sum, we found that research on work-related factors and CLBP has mainly stressed on the role of *task characteristics* (workload, control) and *interpersonal characteristics* (support). However, in line with the AW model it might be valuable to extend this view in future research to the role of *organizational variables* (i.e., reward, values, fairness).

### Moderator analysis

For most of the reported relationships between psychosocial risk factors and CLBP heterogeneity of effect sizes between studies was indicated. Therefore, average relationships should be interpreted with caution. In turn, we conducted a series of moderator analyses to get more insights on factors explaining such between-study variance.

We found a moderating role of samples’ mean age for the relationship between job control and CLBP. Similarly, Zacher and Schmitt [68] point to interaction effects of work related factors and age on occupational well-being. One explanation could be that older workers in contrast to younger ones have higher emotional competencies that are helpful in dealing with such workplace stressors. This concerns, for instance, the regulation of own emotions and understanding others’ emotions which was as supported by a recent systematic review [52].

Skill discretion did not significantly correlate with CLBP. However, we found exposure duration and sex distribution as potential moderator variables affecting this relationship. First, it is possible that employees actively shape their working conditions in sense of job crafting which, in turn, reduces CLBP. Job crafting goes beyond the more traditional ‘top-down’ concepts of work design and describes the active redesign of one’s own work by the employees themselves as a bottom-up process [69, 70]. Through job crafting employees regain control and influence at work [70]. Second, these results further suggest that it is necessary to keep such demographic variables as sex and age (as we discussed above) not only as confounders of CLBP but also as potential moderating variables. Therefore, future studies should compare adjusted models with moderator models (e.g., stratified models) when investigating relationships between psychosocial risk factors and CLBP.

### Limitations

Our review is not without limitations. First, we conducted an extensive literature research of studies. However, the number of available studies for data aggregation was limited. Although the number of studies is similar to other reviews in this research [25, 27, 41], the small number of cases affects the precision of effect size estimates and also the possibility to conduct moderator analyses because of low statistical power. In addition, we note that we were not able to adjust pooled effect size estimates for unreliability and ‘artificial’ dichotomization of variables as information was missing in the studies [46]. Consequently, our results most likely represent rather conservative estimates of true effects. Future research in this domain should report reliability estimates of measures and should use the full-scale range instead of dichotomizing variables.

Second, we included articles from published peer-reviewed journals and only articles in German or English language. By chance, these studies primarily examined Caucasian populations from Europe. Therefore, pooled effect size estimates and heterogeneity of effect sizes might change when including samples from other countries and, in addition, when integrating data from unpublished studies. However, with the relationship between skill discretion and CLBP as an exception, we found only weak evidence for a possible publication bias [46]. In addition, simulation analyses revealed only a minor impact of such a bias for the presented average effects. Thus, the reported pooled effect size estimates seem to be relatively robust. Nevertheless, future meta-analyses might extend the scope of literature search.

Finally, the low to medium quality of included studies might have biased our results. The most common problem involves an unspecific assessment of the outcome. Although CLPB was clearly defined according to our inclusion criteria (pain in the lumbar region lasting for 3 month or longer), many studies did not apply such a measure (see e.g. [60*, 61, 71*]). One reason might be a lack of agreement about the definition of CLBP [16, 17] and, in turn, no consistent use of measures. In addition, some studies did not report the reliability of the instruments to measure psychosocial stressors or main characteristics of the study population. Also, adjustment of confounders varied across the studies. However, we always used effect sizes for pooling that were at least adjusted for demographic variables, also to strengthen their comparability. Moreover, reported pooled effect size estimates were comparable in studies using prospective designs with higher quality and, in addition, we found no evidence that methodological quality of studies was a moderator affecting the reported effect size estimates. In sum, we conclude that although our review of literature calls for more high quality studies in this research, study quality is not a variable explaining the results reported here.

### Research implications

In view of the changes within the current working world, job exposures that shape the exchange and interplay between organization and employee, for instance, reward, fairness, and values, are expected to become more important in maintaining health in general and preventing CLBP in particular [72, 73]. Consequently, there is a need for future research investigating those constructs more specifically. Additionally, we recommend including all of the AW factors [32] that are workload, control, support, reward, fairness, and values. Keeping up this rationale, it would be possible to investigate combined additive and interactive effects of these psychosocial work-related factors over and above the assumptions from the JDCS model [31, 74] and the effort-reward imbalance model [34].

An enormous challenge in preparing the systematic review was the identification of studies using an accurate and rigorous definition of CLBP. We defined CLBP as unspecific LBP lasting for 3 month or longer. During literature search, we noted that there is a substantial lack in studies investigating the association between psychosocial work factors and CLBP following this definition. Future research should use a more consistent and rigorous definition of CLBP, apply appropriate (valid and reliable) measures for CLBP in order to improve consistency of results and to allow a comparative analysis. Meucci and colleagues [75] suggested a minimal definition of CLBP that includes a precise description of the anatomical area, the pain duration, and level of CLBP induced limitations in general daily activities. Moreover, to increase the validity of diagnosis the assessment of CLBP by interviews and by medical examinations should be preferred in contrast to self-report questionnaires.

Although we found a number of prospective studies that could be included in our review, future research should apply high quality randomized and longitudinal case-control studies as well as intervention studies more often. Such designs allow investigating causal interference of relationships between work exposures and CLBP more strongly. Therefore, future research should investigate psychosocial risk factors of the AW model in combination when exploring antecedents of CLBP.

### Practical implications

In view of the rising burden and associated high costs of CLBP [76–78] for the *individuals* (e.g., reduced life activities, impaired well- being), for the *employers* (e.g., lower work performance, higher absence rates from work), and for the *society* (e.g., expenses of health care services and social welfare system) this meta-analysis yields important implications for public health and human resource management. In particular, the chronic state of back pain constitutes a unique clinical syndrome [1] representing a great challenge for interventions [79]. Our results suggest that psychosocial job exposures (workload, control and social support) are not only associated with a higher risk for lower back pain (e.g. [25]) but also with a higher risk that this becomes chronic. Therefore, a reduction of those stressors and the design of healthy job exposures are required for CLBP prevention.

Using a stepwise approach, first, potential risk factors at work have to be assessed with valid instruments, for instance, by self-report [35, 80] or by workplace observation [81, 82]. Second, organizational-level interventions designed to change and to optimize those psychosocial factors (e.g. task restructuring, increasing work control or the level of participation) need to be implemented. More specifically, other research found that if the involvement of employees during interventions is high, measurements focusing on the design of ‘healthy’ workplaces are more successful [83, 84]. For instance, involvement can be increased bottom-up if employees develop context-specific solutions in cooperation, prepare action plans targeting the improvement of their health and well-being, and, in turn, implement and evaluate these measures. There might be situations where a reduction of psychosocial stressors is hardly possible (e.g., high workload because of absence-related understaffing). Therefore, according to our results, it is necessary to strengthen potential job resources with the power to reduce adverse (physical) effects of high job demands [85]. This concerns task-level and interpersonal-level work factors such as time and method control and opportunities for social support but also time to recover from work [86]. For instance, a recent meta-analysis showed that even paid within-shift breaks reduce employees’ physical discomfort and increase their well-being and task-performance [87]. Moreover, increasing employees’ psychological detachment from work seems to be a helpful recovery process for preventing physical discomfort and back pain [86, 88]. In sum, participatory and organizational-focused interventions could serve as an important complement to the widely used individual-level measures [89, 90] to reduce the risk of CLBP.

## Conclusion

In this meta-analysis, we found substantial evidence that psychosocial work factors such as high workload, low job control, and low social support drive risks in developing CLBP. Although our reported effect sizes are rather conservative estimates, undermining potential true effects, the results revealed robust evidence of an association between exposures to work-related psychosocial risk factors and CLBP, even in prospective studies. However, after reviewing the existing literature we also found several challenges that need to be considered in future studies when trying to explain how CLBP is shaped, affected, and to be prevented.

## Data Availability

Meta-analysis data and systematic materials can be requested from authors.

## Appendix 1

### Search string

#### Study population (sensitive string according to Mattioli, 2010 [91]

(occupational diseases [MH] OR occupational exposure [MH] OR occupational exposure* [TW] OR “occupational health” OR “occupational medicine” OR work-related OR working environment [TW] OR at work [TW] OR work environment [TW] OR occupations [MH] OR work [MH] OR workplace* [TW] OR workload OR occupation* OR worke* OR work place* [TW] OR work site* [TW] OR job* [TW] OR occupational groups [MH] OR employment OR worksite* OR industry) AND

#### Outcome

((back pain) OR (low back pain) OR (chronic back pain) OR (chronic* AND back pain) OR (back disorder) OR (back complaints) OR (musculoskeletal disorder*) OR (musculoskeletal disease*)) AND

#### Exposure^1^

((workload) OR (workload) OR (work load[MeSH Terms])) OR (mental workload) OR (mental load) OR (emotional load) OR (occupational workload) OR (physical workload) OR (work demand) OR (job strain) OR (intensity of labour) OR (intensity of labor) OR (work environment)) OR (quantity of work) OR (job demand) OR (job load) OR (capacity) OR (time pressure)) OR ((work control) OR (decision latitude) OR (decision authority) OR (job control) OR (work autonomy) OR (job influence) OR (control[MeSH Terms]))OR (autonomy)) OR ((social support*) OR (support) OR (work conflict) OR (community)) OR ((reward) OR (payment) OR (effort-reward) OR (job opportunit*) OR (promotion prospects) OR (bonus)) OR ((fairness) OR (injustice) OR (justice) OR (equality) OR (unfairness)) OR ((value*) OR (cultur*) OR (standards) OR (ethic*) OR (ideal) OR (principle*) OR (belief*))

## Appendix 2

**Table 7:** Items used to assess the quality of the included studies

Section	In a well conducted study...
A. Study objective / purpose	(1) The study addresses an appropriate and clearly stated question.
B. Study design/ population	(2) The population is selected from a total population that is comparable in all aspects other than the factor under investigation (Positive if the main characteristics of the study population are described; i.e., sampling frame and distribution of the population by age and sex).
C. Exposure assessment	(3) The study assesses and reports all relevant exposures. (4) The study assesses the exposure(s) (psychosocial factors) with valid and reliable instruments.
D. Outcome assessment	(5) The outcomes are clearly defined. (6) The outcome is assessed with reliable and valid instruments. (7) The main potential confounders are identified and taken into account in the study design and data analysis (e.g., individual factors, other psychosocial factors, physical factors).
E. Analysis and data presentation	(8) Effect sizes and their confidence intervals are reported (or can be calculated from other data reported).

Each item was coded as 1 = ‘positive’ or 0 = ‘negative or unclear’. A total sum score was calculated indicating study quality
